# Supplementary figures and images for: A network of cytosolic (co)chaperones promotes the biogenesis of mitochondrial signal-anchored outer membrane proteins
Source: eLife. 2022 Jul 25;11:e77706. doi: 10.7554/eLife.77706 (PMC9355564; doi:10.7554/eLife.77706)

Figure 1-source data 1

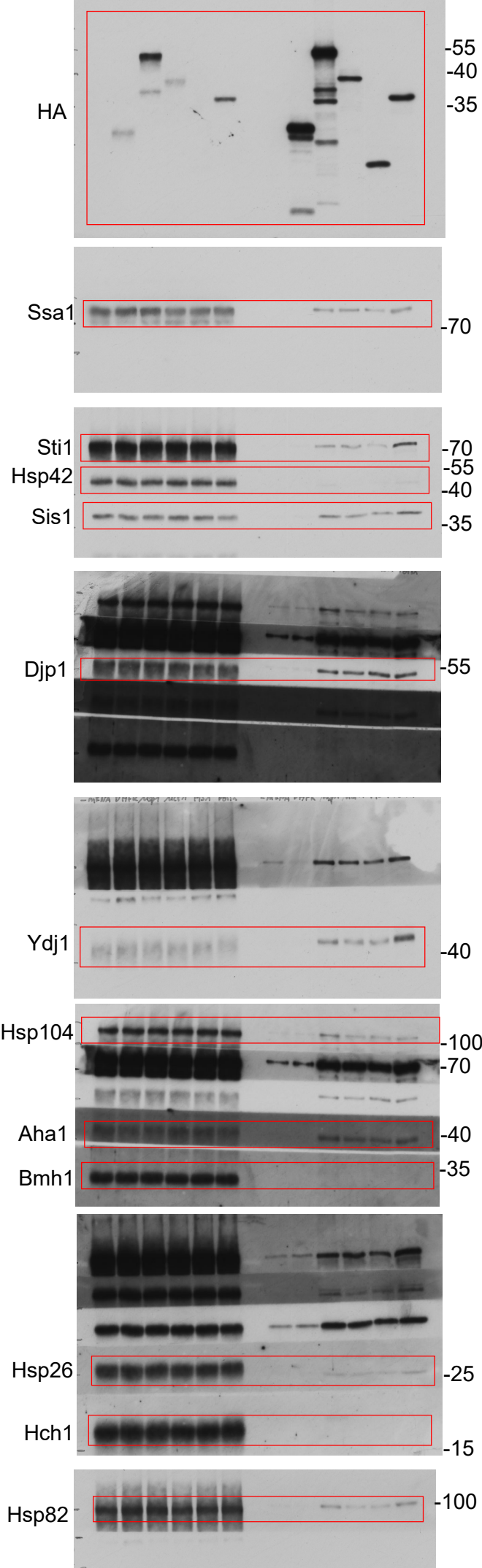

Supplement: Figure 1—source data 1. [file elife-77706-fig1-data1.pdf]

Figure 1-source data 2

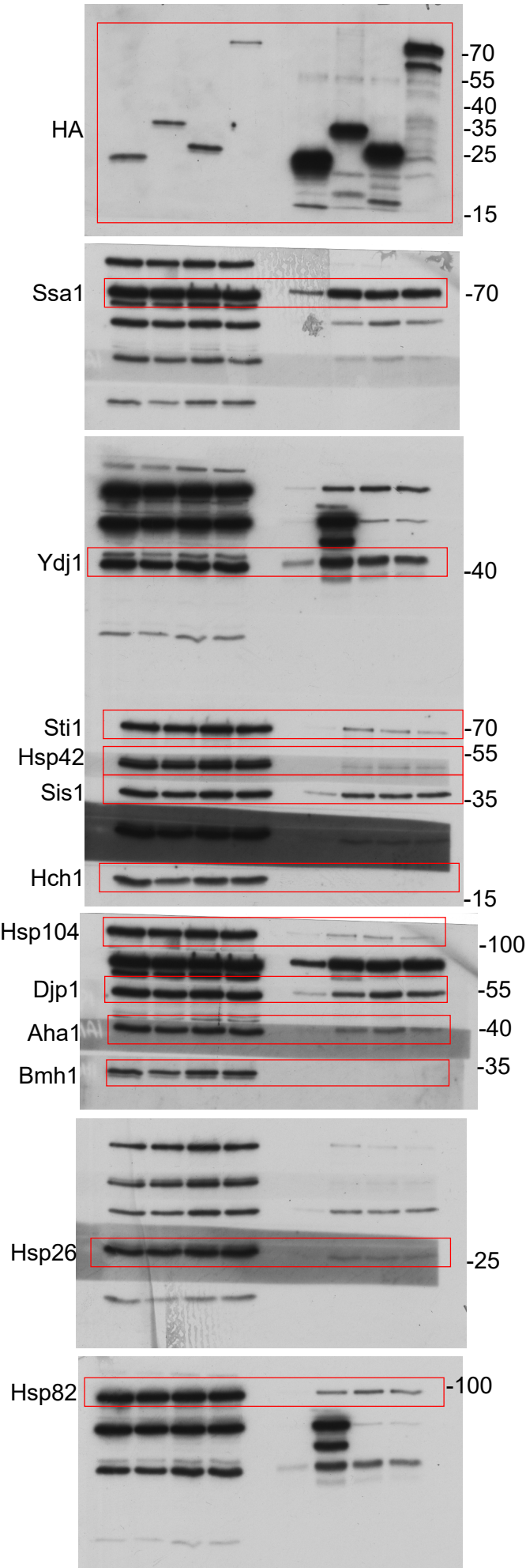

Supplement: Figure 1—source data 2. [file elife-77706-fig1-data2.pdf]

Figure 2-source data 1

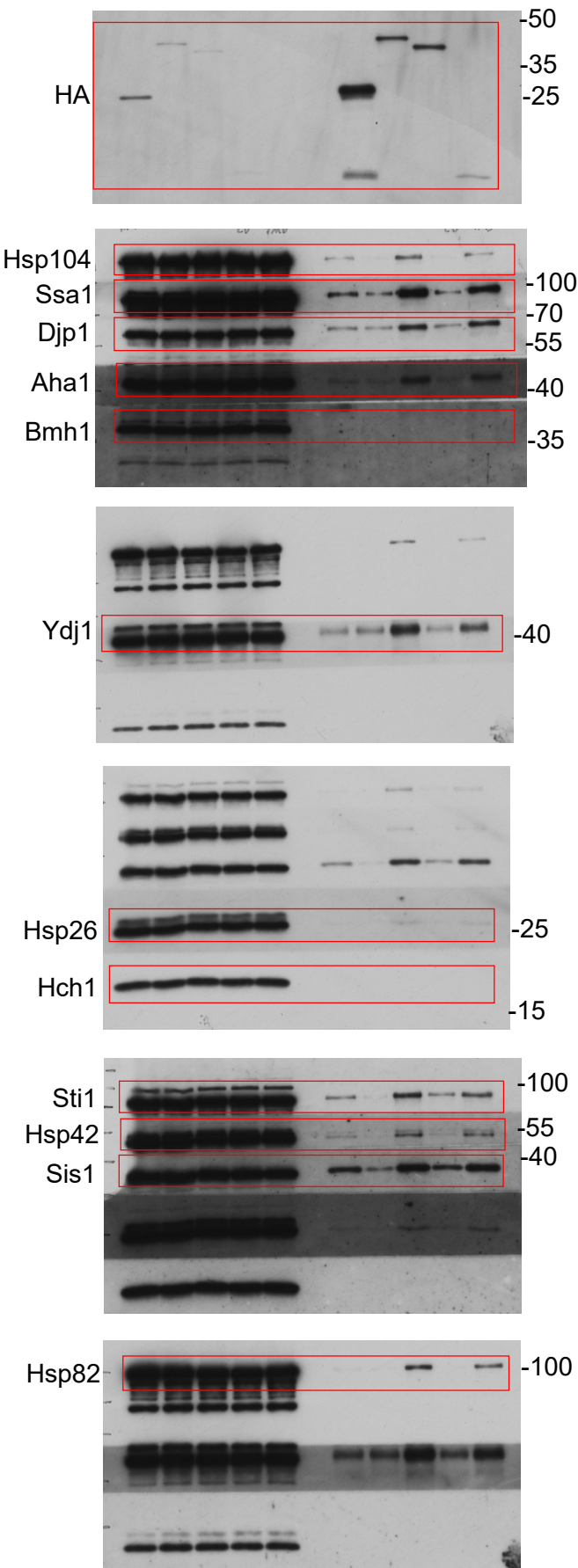

Supplement: Figure 2—source data 1. [file elife-77706-fig2-data1.pdf]

Figure 2-source data 2

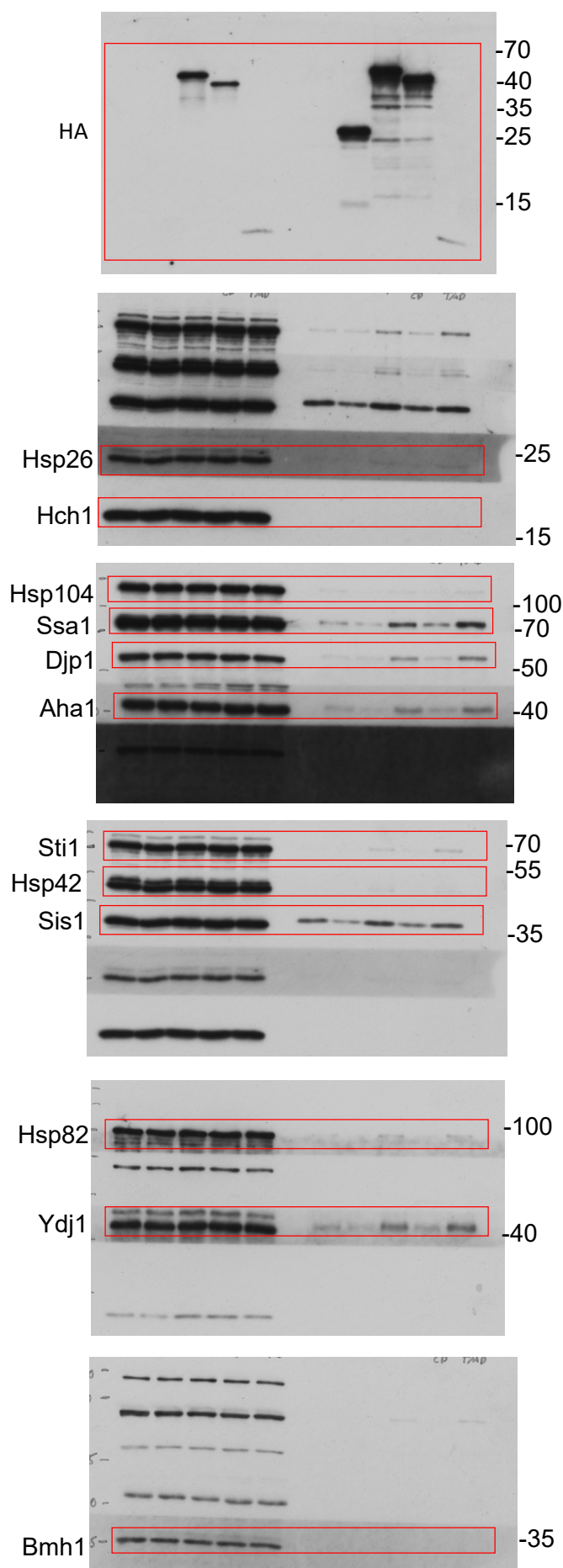

Supplement: Figure 2—source data 2. [file elife-77706-fig2-data2.pdf]

Figure 2-source data 3

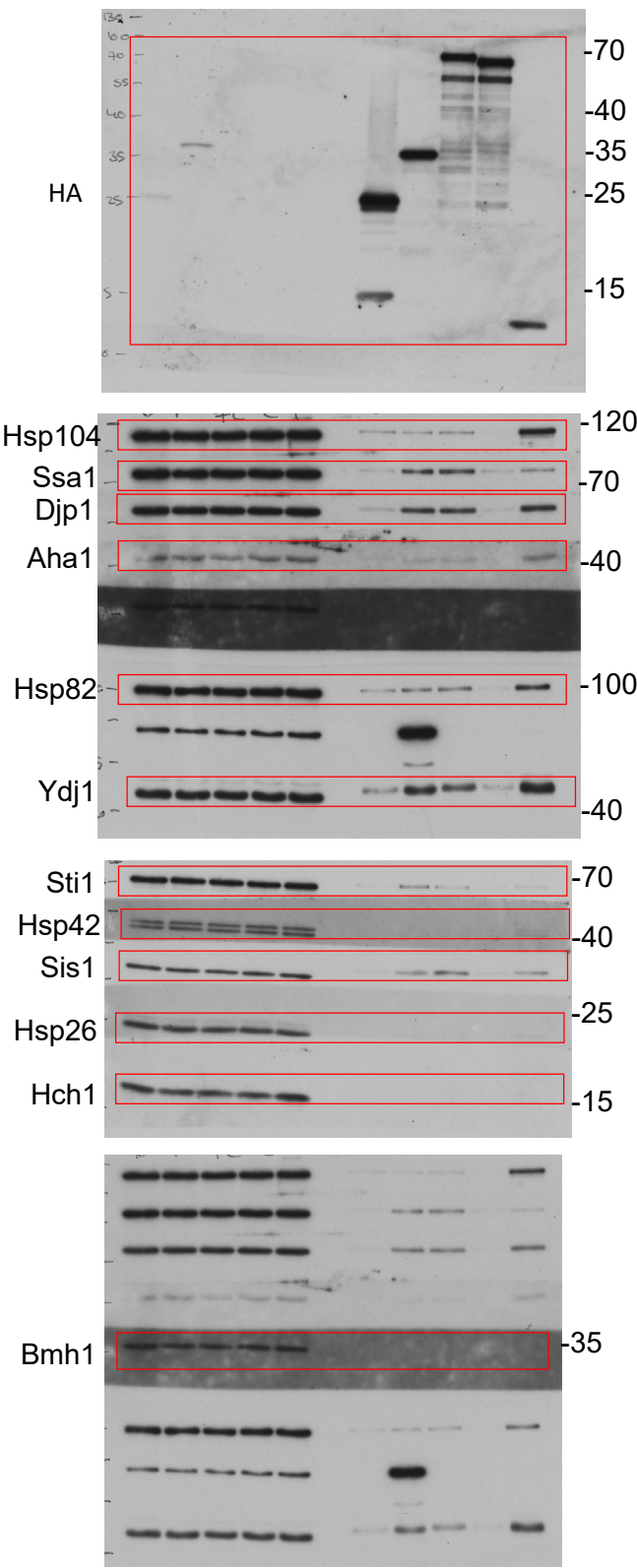

Supplement: Figure 2—source data 3. [file elife-77706-fig2-data3.pdf]

Figure 3-source data 1

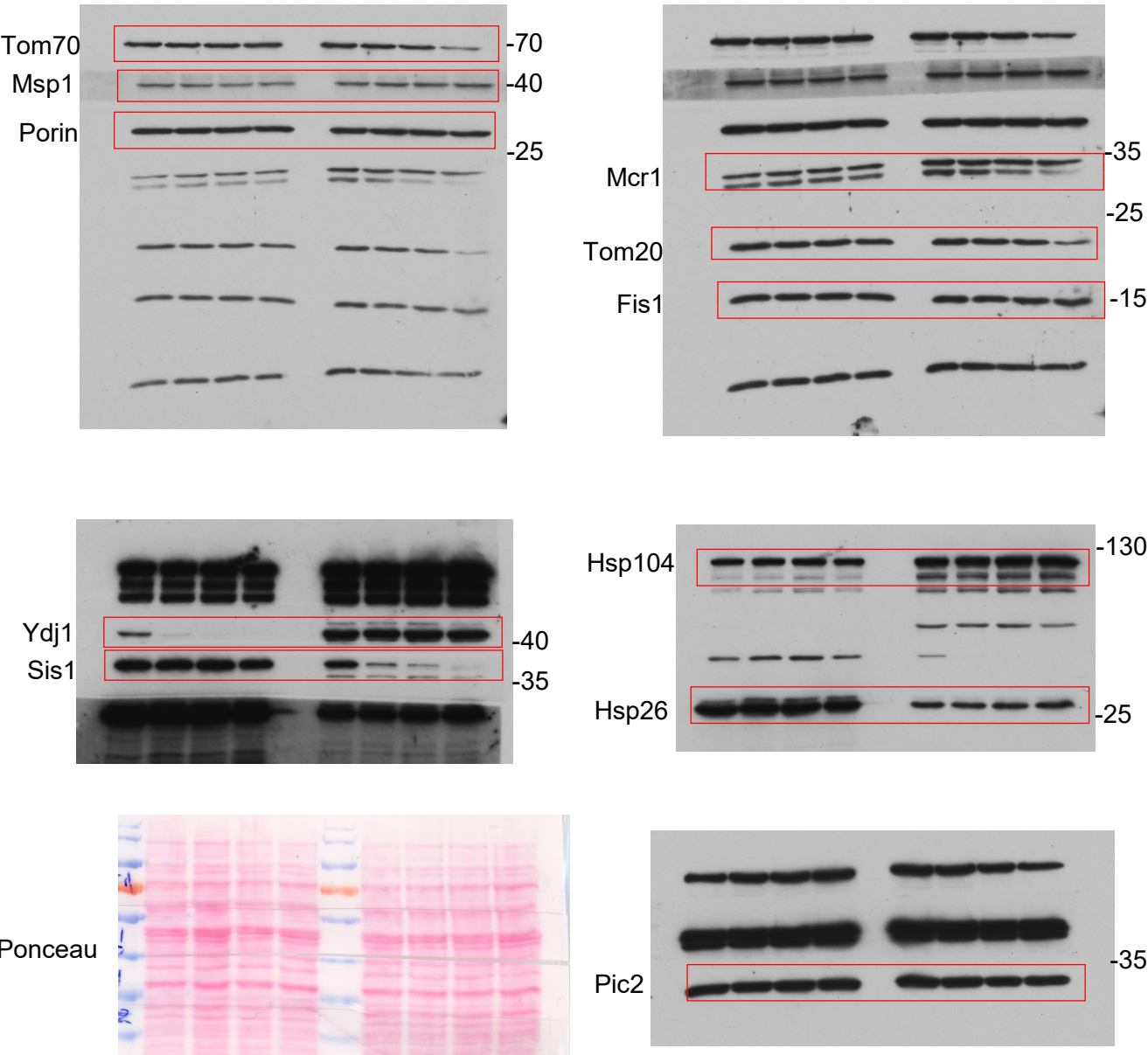

Supplement: Figure 3—source data 1. [file elife-77706-fig3-data1.pdf]

Figure 3-source data 2

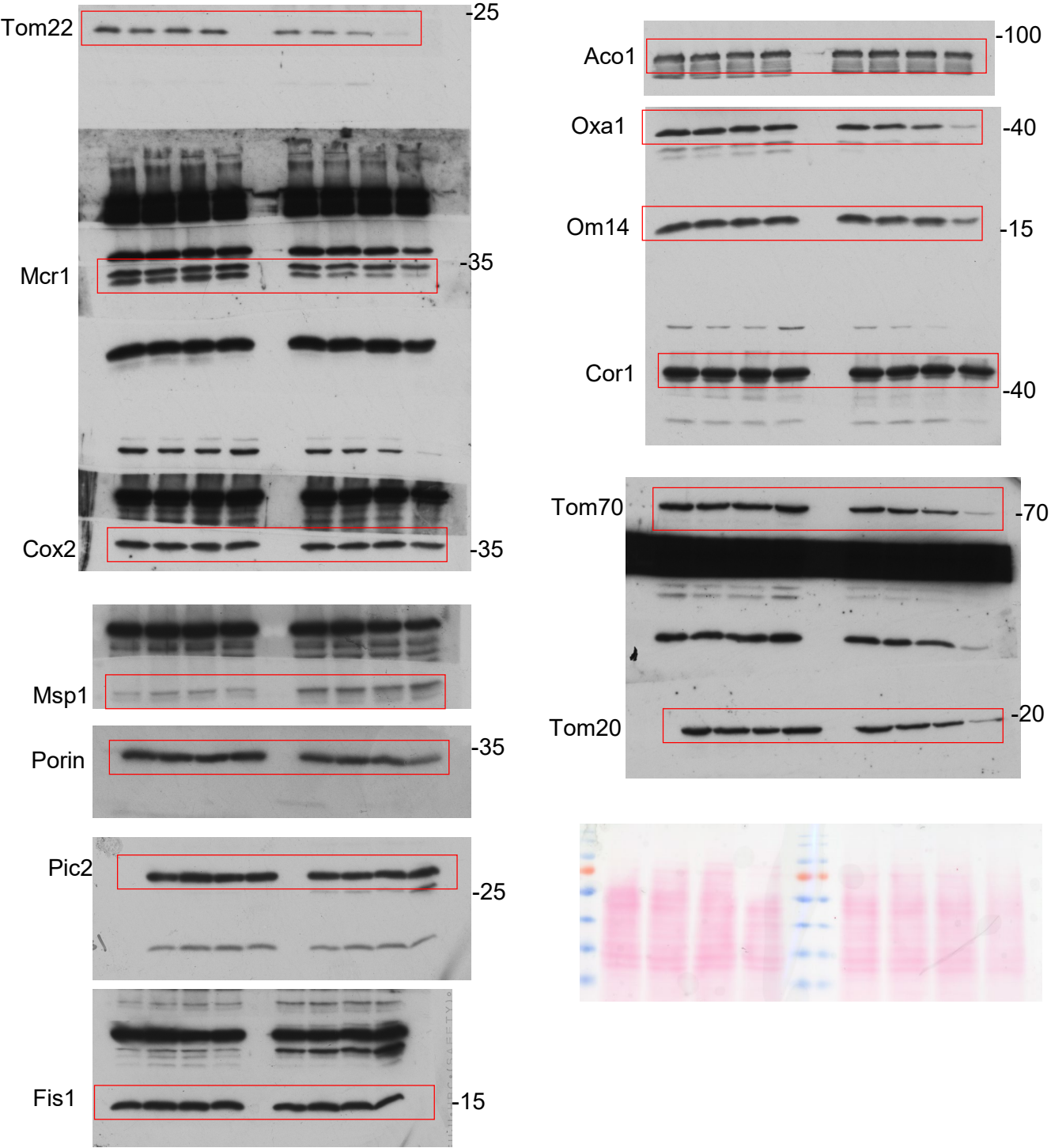

Supplement: Figure 3—source data 2. [file elife-77706-fig3-data2.pdf]

Figure 3-source data 3

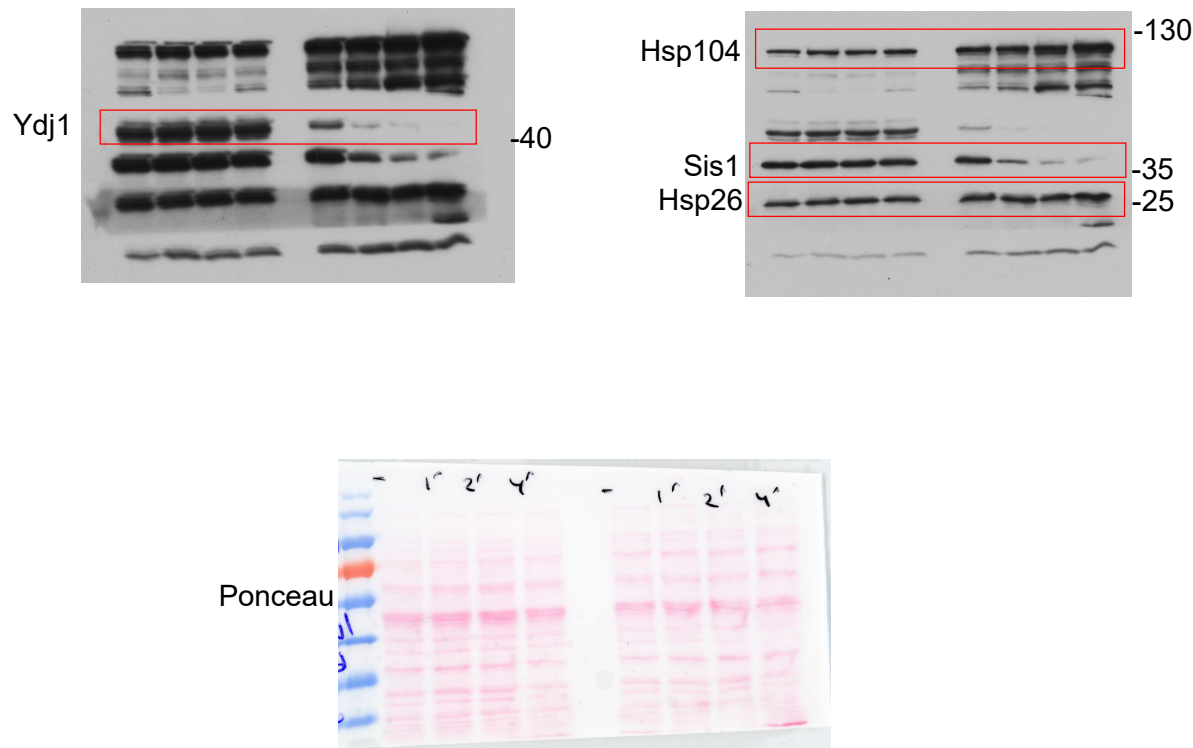

Supplement: Figure 3—source data 3. [file elife-77706-fig3-data3.pdf]

Figure 3- Figure supplement 1-source data 1

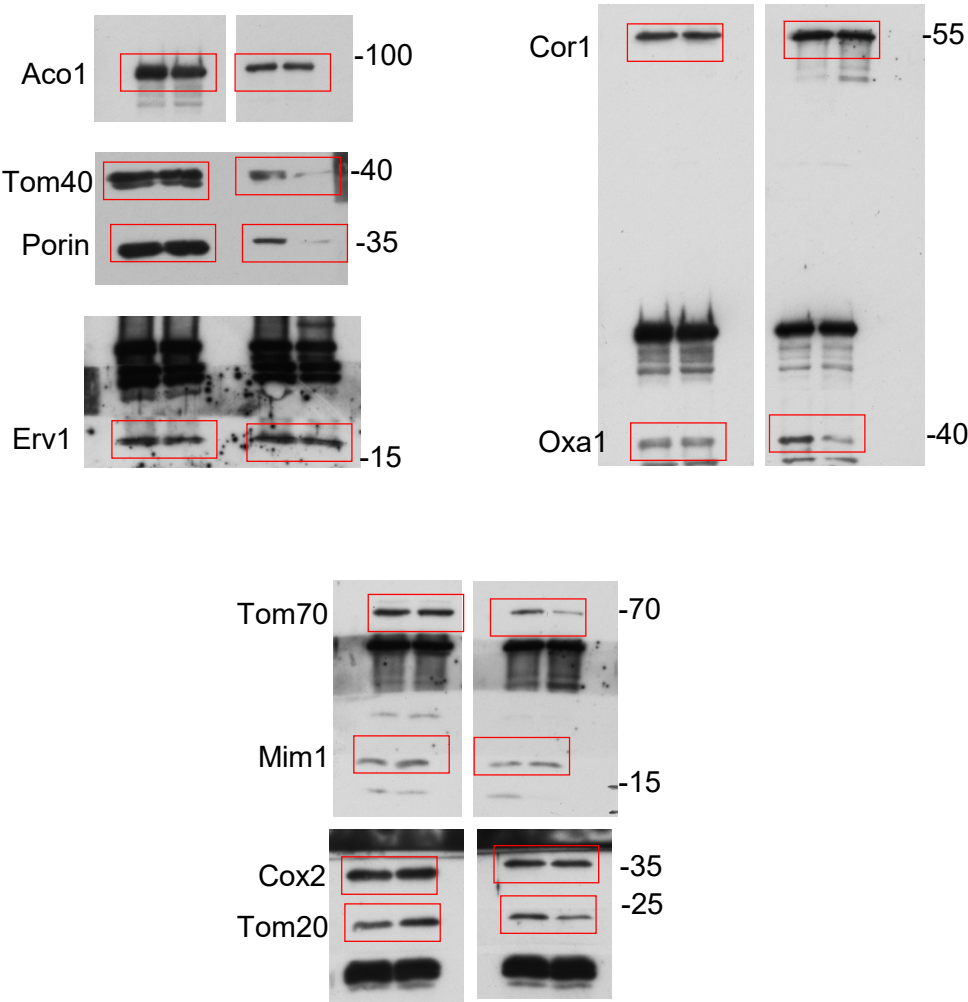

Supplement: Figure 3—figure supplement 1—source data 1. [file elife-77706-fig3-figsupp1-data1.pdf]

Figure 3- Figure supplement 1-source data 2

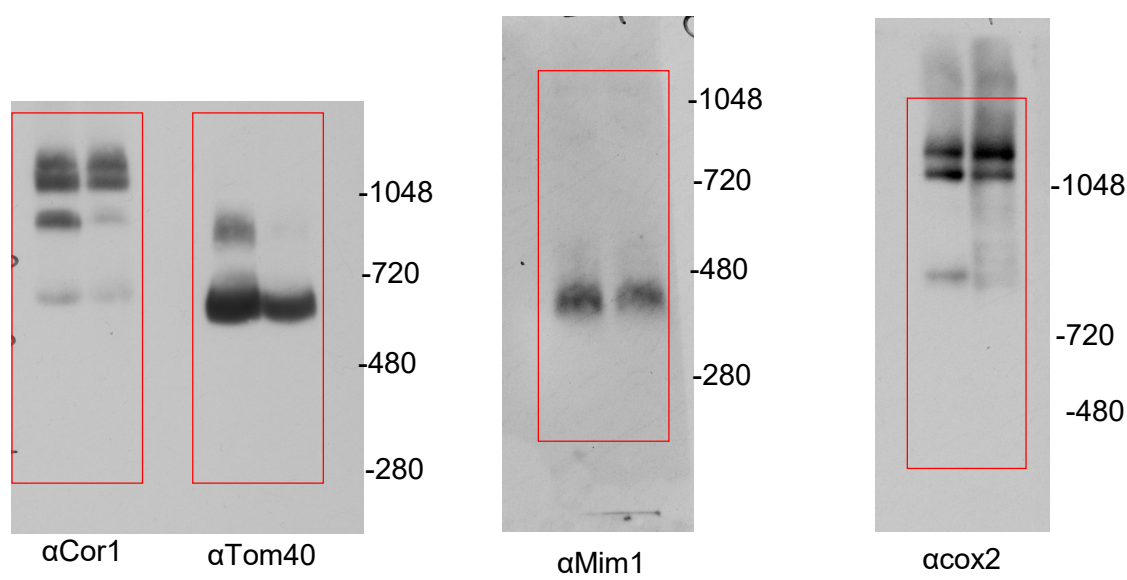

Supplement: Figure 3—figure supplement 1—source data 2. [file elife-77706-fig3-figsupp1-data2.pdf]

Figure 4-source data 1

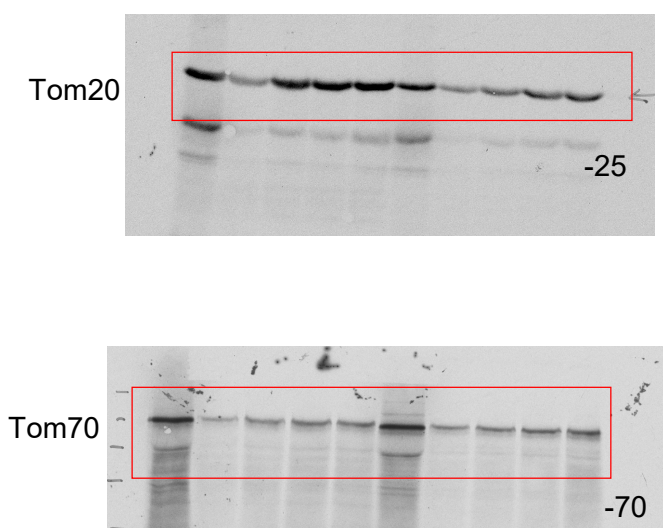

Supplement: Figure 4—source data 1. [file elife-77706-fig4-data1.pdf]

Figure 5-source data 1

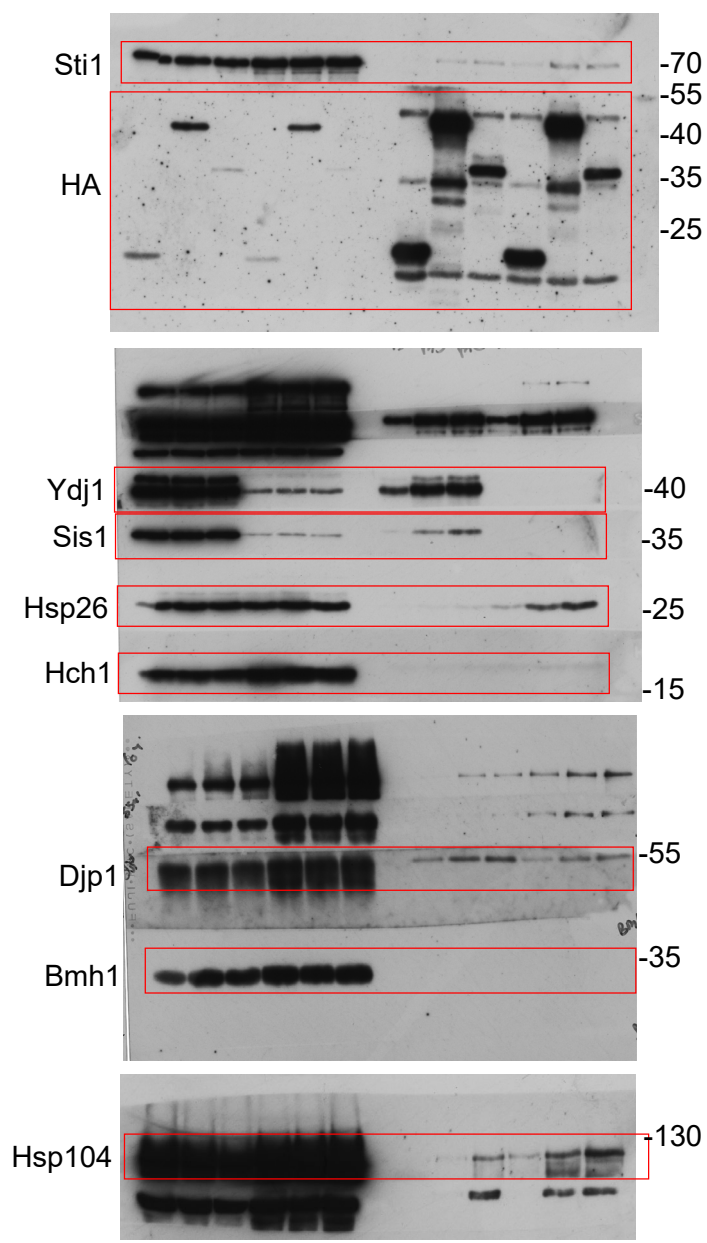

Supplement: Figure 5—source data 1. [file elife-77706-fig5-data1.pdf]

Figure 5-source data 2

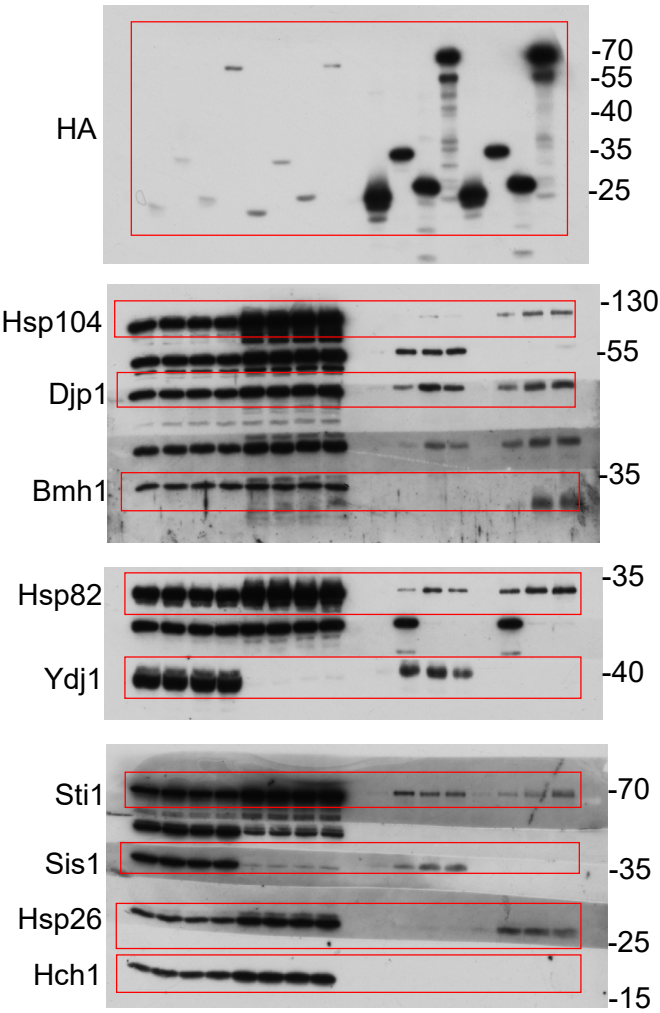

Supplement: Figure 5—source data 2. [file elife-77706-fig5-data2.pdf]

Figure 7-source data 1

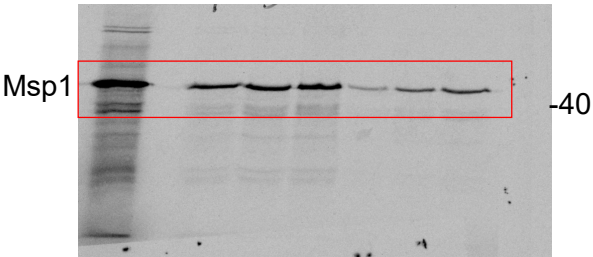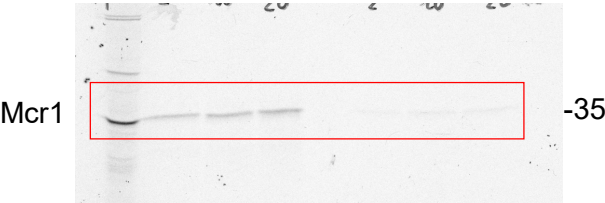

Supplement: Figure 7—source data 1. [file elife-77706-fig7-data1.pdf]

Figure 8-source data 1

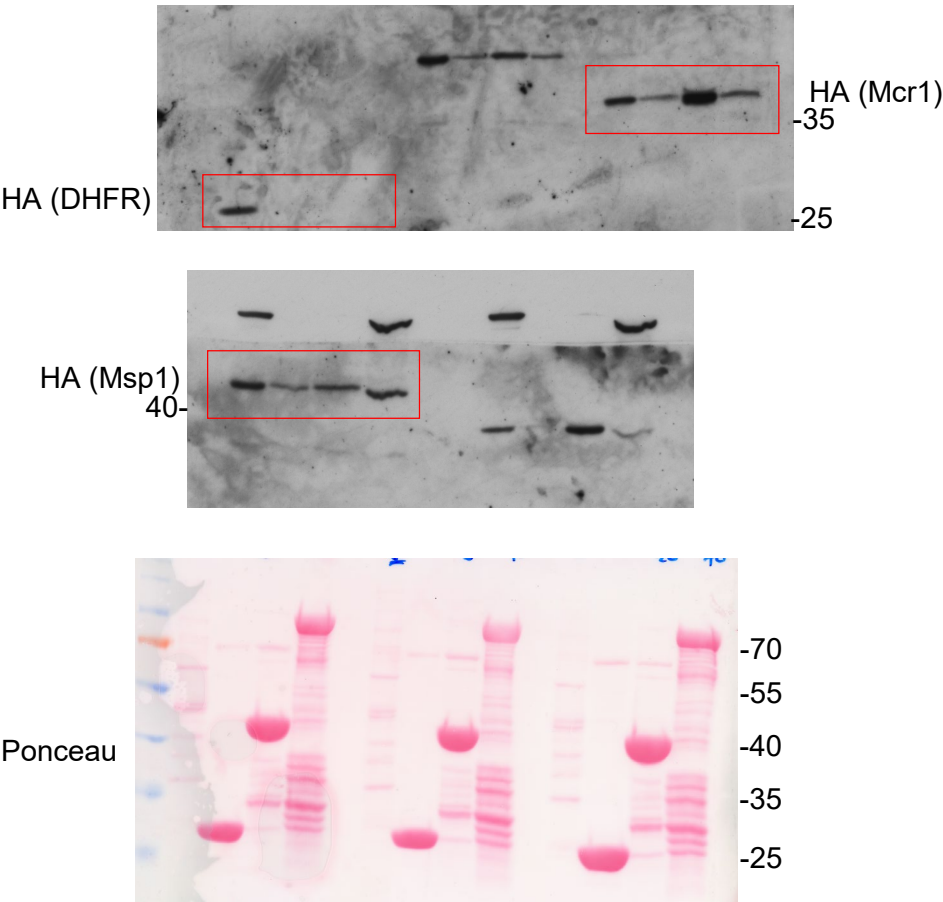

Supplement: Figure 8—source data 1. [file elife-77706-fig8-data1.pdf]

Figure 8- Figure supplement 1-source data 1

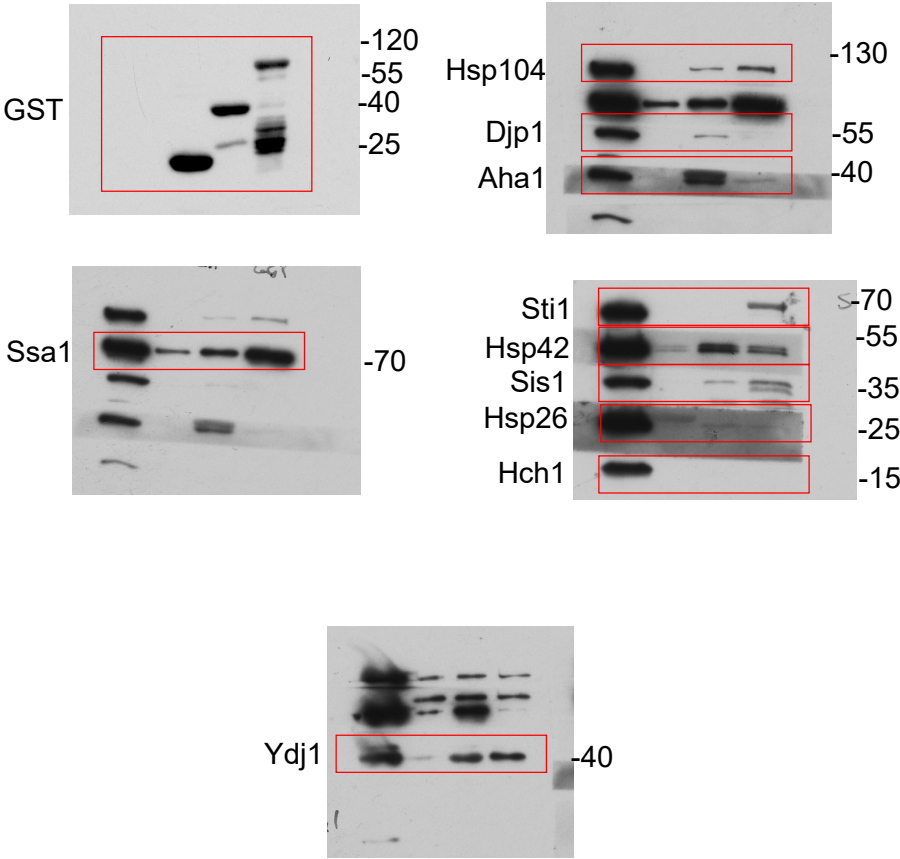

Supplement: Figure 8—figure supplement 1—source data 1. [file elife-77706-fig8-figsupp1-data1.pdf]

Figure 9-source data 1

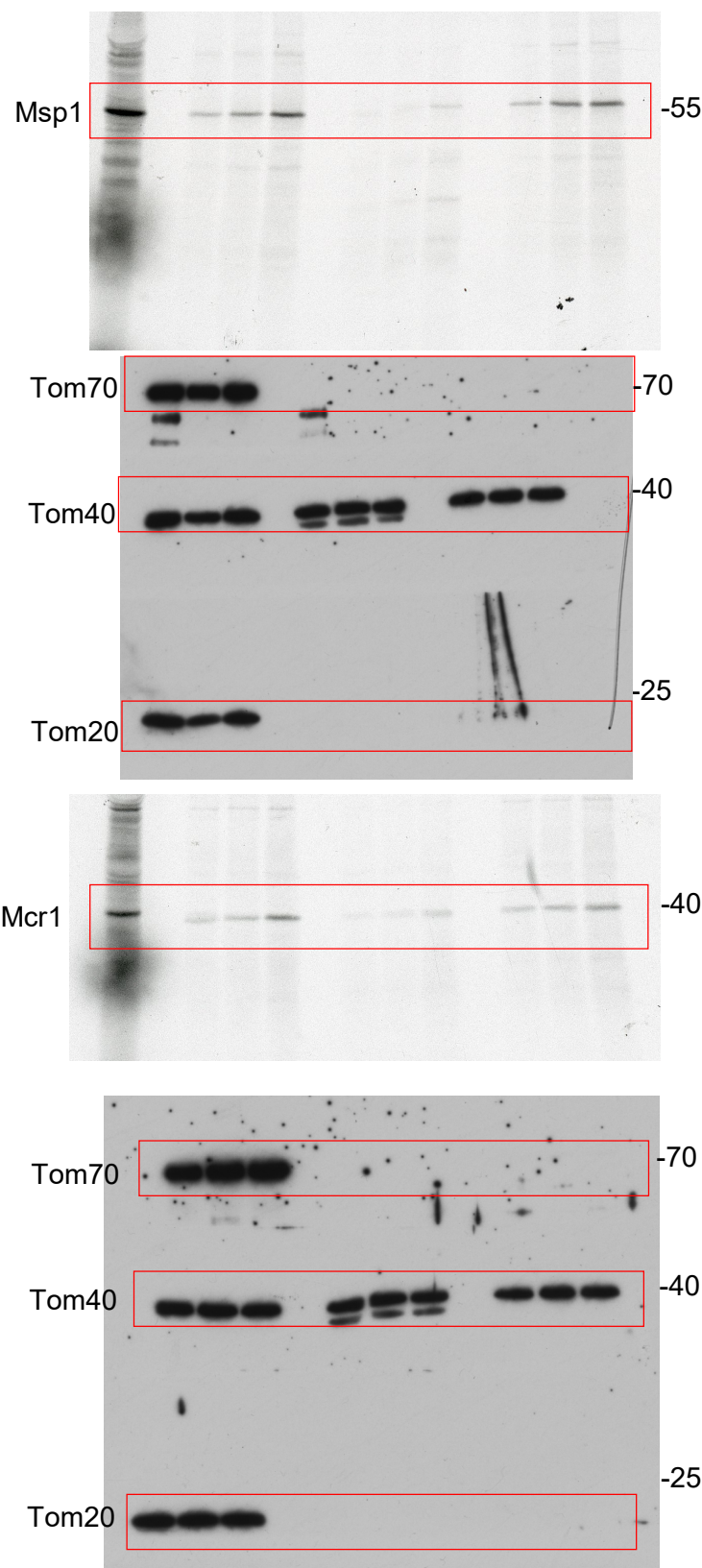

Supplement: Figure 9—source data 1. [file elife-77706-fig9-data1.pdf]

Figure 9-source data 2

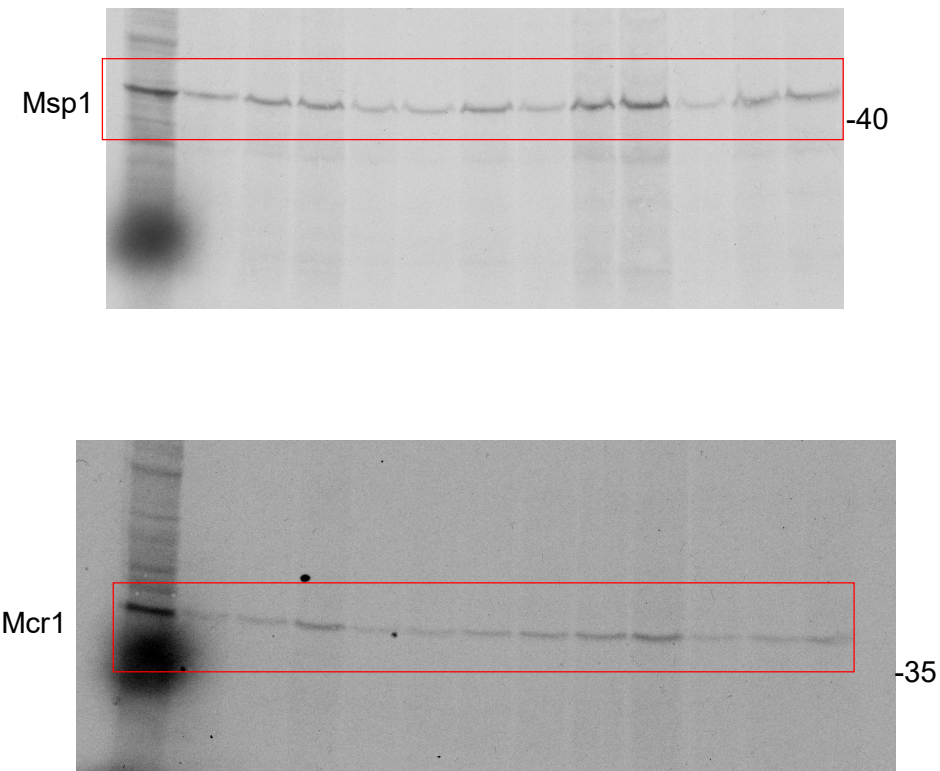

Supplement: Figure 9—source data 2. [file elife-77706-fig9-data2.pdf]

Figure 9- Figure supplement 1-source data 1

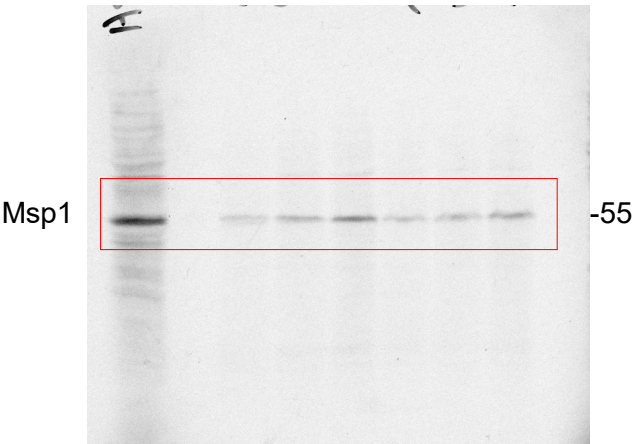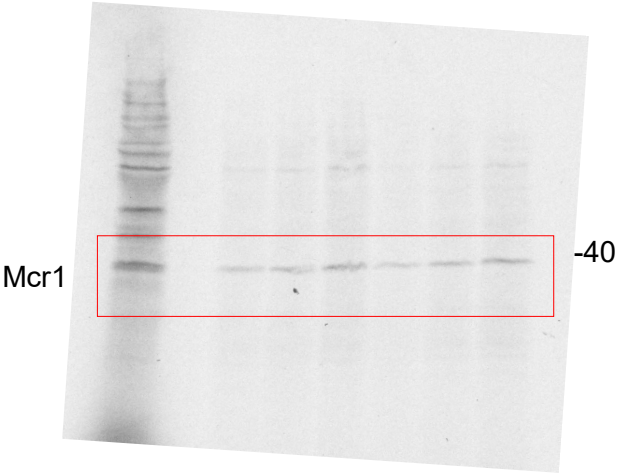

Supplement: Figure 9—figure supplement 1—source data 1. [file elife-77706-fig9-figsupp1-data1.pdf]

Figure 9- Figure supplement 1-source data 2

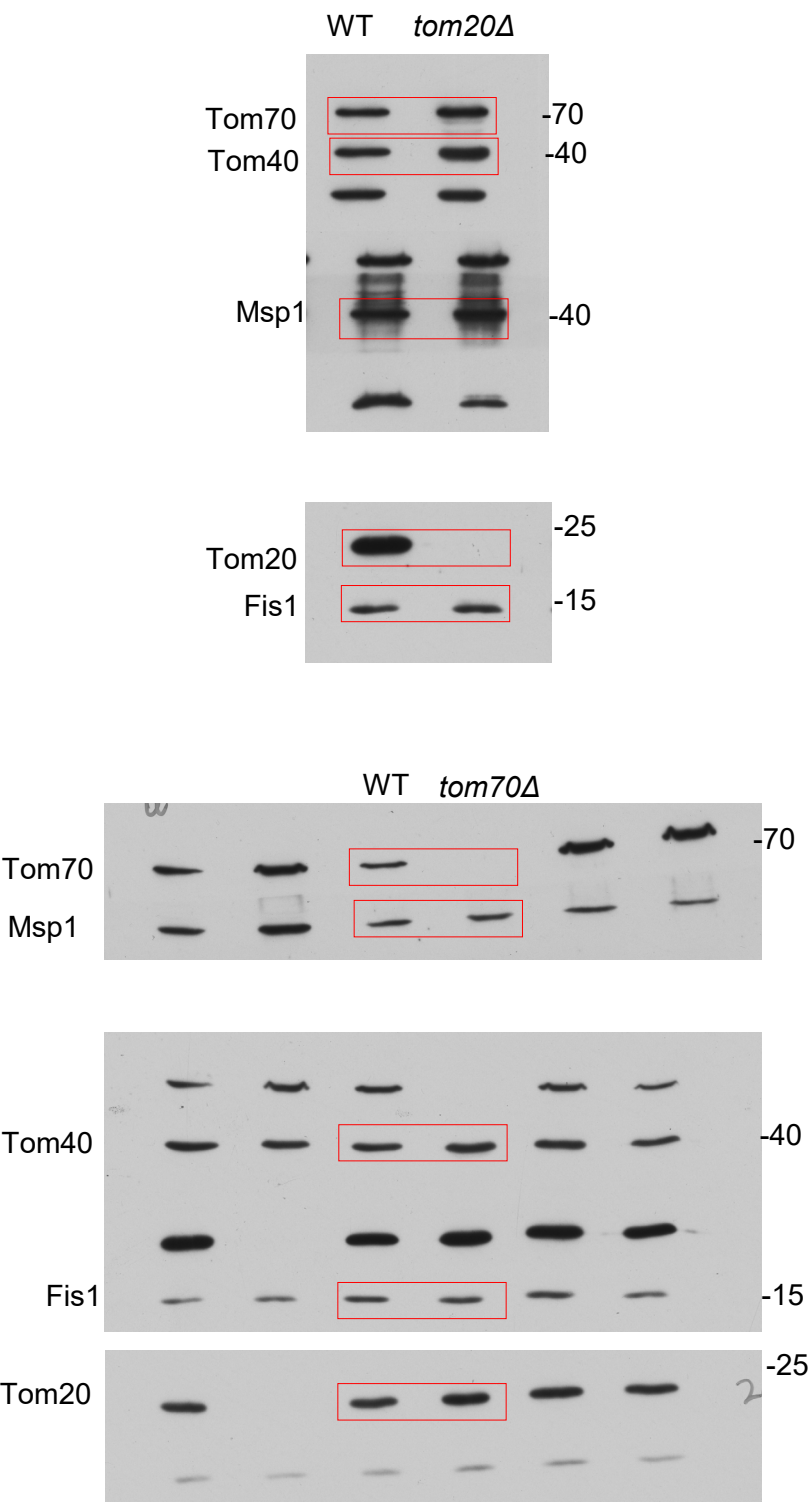

Supplement: Figure 9—figure supplement 1—source data 2. [file elife-77706-fig9-figsupp1-data2.pdf]
