# Supplementary material for: A network of cytosolic (co)chaperones promotes the biogenesis of mitochondrial signal-anchored outer membrane proteins
Source: eLife. 2022 Jul 25;11:e77706. doi: 10.7554/eLife.77706 (PMC9355564; doi:10.7554/eLife.77706)
Supplement: Supplementary file 1. — (A) List of chaperones that were found in the elution fraction of either Msp1 or Mcr1 but not in the elution of mock pull-down (0). The iBAQ values of the indicated proteins are indicated. (B) A list of chaperones that were enriched in the elution fraction of either Msp1 or Mcr1 as compared to their levels in the elution from mock pull-down assay are indicated. The iBAQ value of each protein in the eluate of the mock pull-down was set to 1 and the relative values in the pull-down assays with wither Msp1 or Mcr1 are indicated. [file elife-77706-supp1.docx]

**Supplementary File 1: (co)chaperones that co-purified with *in vitro* translated proteins**

**A**

| **Protein name** | **Gene name** | **Mock** | **Msp1 (iBAQ)** | **Mcr1 (iBAQ)** |
| --- | --- | --- | --- | --- |
| Heat shock protein STI1 | STI1 | 0 | 1383200 | 6860600 |
| Vacuolar transporter chaperone 4 | VTC4 | 0 | 1575800 | 3677300 |
| Vacuolar transporter chaperone 2 | VTC2 | 0 | 270840 | 2372800 |
| Mitochondrial clpX-like chaperone MCX1 | MCX1 | 0 | 719400 | 2178900 |
| ATP-dependent molecular chaperone HSP82 | HSP82 | 0 | 180250 | 1771700 |
| HSP70 co-chaperone SNL1 | SNL1 | 0 | 171400 | 1531500 |
| Heat shock protein SSA4 | SSA4 | 0 | 0 | 828380 |
| Heat shock protein 70 homolog LHS1 | LHS1 | 0 | 178030 | 384110 |
| SEC14 cytosolic factor | SEC14 | 0 | 356900 | 0 |

**B**

| **Protein name** | **Gene name** | **Mock** | **Msp1**  **(relative iBAQ)** | **Mcr1**  **(relative iBAQ)** |
| --- | --- | --- | --- | --- |
| Hsp90 co-chaperone AHA1 | AHA1 | 1 | 59.2 | 70.1 |
| Heat shock protein SSB2 | SSB2 | 1 | 6.6 | 17.6 |
| ATP-dependent molecular chaperone HSC82 | HSC82 | 1 | 8.6 | 16.1 |
| Heat shock protein SSA2 | SSA2 | 1 | 3.5 | 11.0 |
| Heat shock protein SSC1, mitochondrial | SSC1 | 1 | 4.9 | 9.7 |
| Heat shock protein 104 | HSP104 | 1 | 4.7 | 9.4 |
| Heat shock protein SSA1 | SSA1 | 1 | 3.3 | 8.7 |
| Heat shock protein 60, mitochondrial | HSP60 | 1 | 14.9 | 8.0 |
| Heat shock protein homolog SSE1 | SSE1 | 1 | 3.0 | 7.6 |
| Heat shock protein SSB1 | SSB1 | 1 | 1.8 | 4.7 |
| Mitochondrial protein import protein MAS5 | YDJ1 | 1 | 4.0 | 2.8 |
| Protein SIS1 | SIS1 | 1 | 1.4 | 2.0 |
| Zuotin | ZUO1 | 1 | 1.50 | 1.87 |

**Table S1**: (A) List of chaperones that were found in the elution fraction of Msp1 and Mcr1 but not in the elution of mock pull-down. The iBAQ values of the specified proteins are indicated. (B) List of chaperones that were enriched in the elution fraction of Msp1 and Mcr1 as compared to their levels in the elution from mock pull-down assay are indicated. The iBAQ value of each protein in the eluate of the mock pull-down was set to 1 and the relative values in the pull-down assays with either Msp1 or Mcr1 as compared to the value in the mock sample are indicated.
